# Prevalence of Oral Mucosal Lesions in an Adult Iranian Population

**DOI:** 10.5812/ircmj.4608

**Published:** 2013-07-05

**Authors:** Fariborz Mansour Ghanaei, Farahnaz Joukar, Maryam Rabiei, Alireza Dadashzadeh, Ali Kord Valeshabad

**Affiliations:** 1 Gastrointestinal and Liver Diseases Research Center (GLDRC), Guilan University of Medical Sciences, Rasht, IR Iran

**Keywords:** Mouth Mucosa, Adult, Iran

## Abstract

**Background:**

Nowadays the importance of oral health to life quality is not obvious to anyone in our world. Oral lesions can interfere with daily social activities in involved patients through impacts on mastication, swallowing and speech and symptoms like xerostomia, halitosis or dysesthesia.

**Objectives:**

To assess the prevalence and types of oral lesions in a general population in Rasht, Northern Province of Iran.

**Patients and Methods:**

1581 people aged > 30 years old who were inhabitant of Rasht, Iran, enrolled in a cross-sectional study. For each individual a detailed questionnaire based on the world health organization (WHO) guidelines in order to diagnosis of the lesions was filled and it contained all the required data. Participants were divided into two groups with and without oral mucosal lesions and oral mucosal lesions were divided into two groups with and without. Demographic characteristics and clinical information including age, sex, smoking (cigarette and tobacco), opium consumption, medication and oral and dental hygiene were collected and compared between these two groups.

**Results:**

The prevalence of mucosal lesions in our study was 19.4%. Our data demonstrated higher prevalence of oral mucosal lesions in males and young adults (30-40 years). The most common mucosal lesion among our participants was Fissured tongue(4%), followed by Fordyce granules(2.8%), geographic tongue(2.6%) , Pigmentation(2.5%), Candida(1.8%), Smoker Plate(1.6%), lingual Varices(1.5%), Petechiae(1.4%) and lingual labial(1.4%) . Leukoplakia was observed only in two people (0.1%).No case of malignant lesions was detected. No statistically significant difference was confirmed between the two groups regarding smoking, opium consumption, medication and oral and dental hygiene.

**Conclusions:**

Our data has provided baseline information about epidemiologic aspects of oral mucosal lesions which can be valuable in organized national program targeting on oral health and hygiene in the society.

## 1. Background

Nowadays the importance of oral health to life quality is not obvious to anyone in our world. Oral lesions can interfere with daily social activities in involved patients through impacts on mastication, swallowing and speech and symptoms like xerostomia, halitosis or dysesthesia **(1)**. Oral cavity was classified as one of the most common ten malignancies from 1975 to 2006 **(2)**. High risk habits such as alcohol and tobacco consumption have been recognized as defined causes for oral precancerous or cancerous lesions **(3, 4)**. Despite the lack of definite evidence supported in the literatures, some dental factors such as improperly fitting denture, electrogalvanism, edentulism, sharp teeth, mouthwashes and poor oral hygiene have been announced as probable causes of oral precancerous and cancerous lesions **(5, 6)**. Epidemiological assays declare a wide variety in the prevalence and most common types of oral lesions in various regions of the world. The prevalence of these lesions in general population has been reported 9.7% in Malaysia **(7)**, 15.5% in Turkey **(8)**, 25% in Italy 4 and 61.6% in Slovenia **(9)**. These lesions have been found in 15% of Saudi Arabian **(10)** and 41.2% of Indian **(11)** dental patients.

The extracted data from these oral health surveys are essential for preparing health strategies in the community. Furthermore, to our knowledge except one published study with our group about status of oral lesions and dental disorders in elderly people **(12)**, there is no other study about the epidemiology of all oral lesions in Iran and most of the conducted studies included only tumors and ulcers or biopsy specimens or just some of the oral lesions in dental patients **(13, 14)**. There is a great sense of needs in Iran for establishing a baseline set of data toward the prevalence of oral lesions in general population.

## 2. Objectives

This study is designed to assess the prevalence and types of oral lesions in a general population in Rasht, Northern Province of Iran.

## 3. Patients and Methods

In this cross-sectional study, 1581 people aged > 30 years who were inhabitants of Rasht, Iran were enrolled in the study. Multisession cluster method was applied for sampling from general population in this city. For performing the research, 2 trained health specialists as interviewers went to people’s houses and explained the objectives of the survey for people aged>30 years old ,in the case of agreement for participation a card for referring to the supposed dental clinic for more examination was given to them. Each participant gave informed consent before enrolling in the study in clinic. Those without an informed consent were excluded from the study. A detailed questionnaire based on the world health organization (WHO) guidelines was filled by the interviewers for each individual which contained all the required data in order to diagnosis of the lesions.

Selected participants underwent complete clinical oral examination by three general dentists who were trained by dental specialist with an intra-examiner agreement upper than 95% and all the information about the type of the lesions and their location (Gingiva, lip, tongue or oral mucosal tissue) were recorded in the questionnaire. Participants were divided into two groups with and without oral mucosal lesions. Oral lesions were divided into two groups: 1) white color lesions including leukoplakia, Leukoedema, lichen planus, smokers` plate, Frictional Keratosis, Candida, Fordyce granules, traumatic ulcers and recurrent aphthous; 2) nonwhite lesions of oral mucosa including herpes labial, Fissured Tongue, geographic tongue, , Hairy tongue, Periapical cyst, Infected tooth related cyst, Pigmentation, Lingual Varices, and Petechiae. Those lesions that could not be diagnosed by clinical examination were just analyzed through histopathological study. A specific code and card were designed for patients with white color lesions that they underwent a histopathological study and these patients were told to return for their final diagnosis based on the biopsies. Demographic characteristics and clinical information including age, sex, smoking (cigarette and tobacco), opium consumption, medication and oral and dental hygiene (use of toothbrush, toothpick, dental floss, mouthwashes, number of filled or decayed tooth) were collected and compared between these two groups. The study protocol was approved by ethics committee of Gastrointestinal and Liver Diseases Research Center of Guilan University of Medical Sciences.

### 3.1. Statistical Analysis

The Kolmogorov-Smirnov test (KS-test) was assigned to assess the normal distribution of the data. Results were reported as mean ± standard deviation (SD) for quantitative variables and percentages for categorical variables. The groups were compared using the Student’s t- test for continuous variables and the chi-square test (or Fisher’s exact test if required) for categorical variables. P values of 0.05 or less were considered statistically significant. All the statistical analyses were performed using SPSS version 16 (SPSS Inc, Chicago, IL, USA) for Windows.

## 4. Results

From 1581 individuals who were enrolled in the study, 306 people (19.4%) had 416 (26.3%) different types of oral lesion. One or more oral mucosal lesions were found in 306 people. Demographic characteristics and baseline clinical data of the participants and their comparisons between two groups are summarized in Table 1. Table 2 shows the frequency and prevalence of various types of oral lesions in our people. The places of these detected lesions are shown in Table 3.

**Table 1.** Demographic Characteristics and Clinical Data of the Participants

Variables	With Oral lLsion (n = 306)	Without Oral Lesion (n = 1275)	P Value
**Male gender**	171 (55.9)	668 (52.4)	0.27
**Mean age (year)**	42.4±13.7	40.9±11.5	0.71
**Age groups**			
< 40	165 (53.9)	671 (52.6)	
40-50	67 (21.9)	356 (27.9)	
50-60	38 (12.4)	160 (12.5)	0.00
60-70	22 (7.2)	68 (5.3)	
> 70	14 (4.6)	20 (1.5)	
**Denture**	83 (27.1)	371 (29.1)	0.49
Tooth decay	135 (44.1)	544 (42.7)	0.65
Filled tooth	194 (63.3)	820 (64.3)	0.77
Toothbrush	272 (88.9)	1146 (89.9)	0.61
Toothpick	69 (22.5)	323 (25.3)	0.32
Dental floss	102 (33.3)	411 (32.2)	0.71
Mouthwash	19 (6.2)	77 (6.0)	0.91
**Medication**	79 (25.8)	304 (23.8)	0.45
Cigarette smoking	44 (14.4)	178 (14.0)	0.85
Tobacco smoking	13 (4.2)	45 (3.5)	0.55
Opium consumption	7 (2.3)	28 (2.1)	0.92

**Table 2.** Frequency and Prevalence of Oral Mucosal Lesion

White color lesion	Frequency, No. (%) (N = 416) ^a^	Prevalence, % (N = 1581)	Nonwhite color lesion	Frequency, No. (%) (N = 416)	Prevalence, No. % (N = 1581)
**Fordyce granules**	44(10.6)	2.8	Fissured tongue	64(15.3)	4
**Candida**	29(6.9)	1.8	Geographic tongue	41(9.9)	2.6
**Smoker plate**	26(6.3)	1.6	Pigmentation	39(9.4)	2.5
**Stomatitis recurrent aphthous**	21(5)	1.3	Lingual Varices	24(5.8)	1.5
**Frictional keratosis**	14(3.4)	0.9	Petechiae	22(5.3)	1.4
**Oral lichen planus**	12(2.9)	0.7	Herpes labial	22(5.3)	1.4
**Leukoedema**	8(1.9)	0.5	Hairy tongue	14(3.4)	0.9
**Leukoplakia**	2(0.5)	0.1	Periapical cyst	7(1.7)	0.4
			Infected tooth related cyst	4(0.9)	0.2

^a^ There were 416 oral lesions in 306 people

**Table 3.** Site of Oral Mucosal Lesions Among 1581 Participants

Site of the lesion	N = 416(%)
**Tongue**	158 (10)
**Gingiva**	87 (5.5)
**Lip**	60 (3.8)
**Oral base**	51 (3.2)
**Soft palate**	26 (1.6)
**Buccal mucosa**	16 (1.01)
**Sublingual**	12 (0.8)
**Vestibule**	4 (0.3)
**Ridge**	2(0.1)

Malignant lesions weren’t found in any participants, either squamous cell carcinoma or adenocarcinoma. Family history of oral cancer, tongue cancer, soft plate and oral cavity were found in 2 (0.1%), 4 (0.3%), 2 (0.1%) and 1 (0.1%) of the participants. It is necessary to mention all of them had no oral lesions.

## 5. Discussion

Despite the considerable prevalence of oral mucosal lesions and their subsequent morbidity in the involved patients, there is no large scaled population-based study regarding prevalence and pattern of these lesions in the society. Our data demonstrated higher prevalence of oral mucosal lesions in males (55.8%) and young adults (30-40 years) (53.9%). However in some studies their prevalence has been found to be higher in older individuals (15, 16) and females (10).Across different regions of the world the frequency and types of oral soft tissue lesions vary. The prevalence of oral mucosal lesions in our study was 19.4% which was considerably lower than what Rabiei M et al. found in institutionalized elderly people in Rasht, Iran (12), and some other studies in other countries (9, 11)**, but it is interesting to know that it was higher than their prevalence in some other countries (2, 7, 8, 10). The most common mucosal lesion among our participants was fissured tongue (4%), followed by Fordyce granules (2.8%), geographic tongue (2.6%) and pigmentation (2.5%). This finding is in consistence with study by Dos Santos et al. (17), however other investigations have reported Fordyce granules (9), melanin pigmentation (18), fibrous dysplasia (19), varices (16) and coated tongue6 as the most common oral lesions.

Tongue lesions possessed a considerable proportion of oral mucosal lesions with different prevalence rate in various parts of the world (16, 20, 21). In our study tongue lesions were observed in 10% of the participants which were higher than previously published study (2, 6, 8, 10). The prevalence of fissured tongue as a common tongue condition is ranges from 5.2% among Turkes (18), 5.7%% among the Indians (11), 21% among the Slovenians (9), 27.5% in the Amazonians (17) and 28% among elderly in This (16). Fordyce granules were found in 2.8% of our patients while in a study by Kovoc-Kovocic and Skaleric in Slovenia it was found as the most common oral condition by a considerable prevalence (49.7%) **(9)**. this lesion was found common in dental patients in Saudi Arabia (3.8%) **(2, 10)** And southern India **(11)**, while it was not so common in Iranian elderly people **(12)**. The third and fourth common oral lesions among our participants were geographic tongue and pigmentation. In an assessment of 243 Spanish children, geographic tongue (4.48%) found as third common lesions of the oral mucosa subsequent subdural tongue (16.02%) and traumatisms (12.17%) (20). Two other studies reported a high frequency for geographical tongue in the north of Iran (13, 14). Mumcu G et al. in a study in Turkey confirmed melanin pigmentation as the most common mucosal lesions in Turkish people (18).

Leukoplakia was observed only in two people (0.1%) in our study which it was considerably lower than the expected range of 1% to 5% (22) also the prevalence rate in other studies was low (2, 10, 11) while the prevalence of lichen planus (0.7) was in the estimated range of 0.1% to 2.2% for general population (23). In an agreement with population-based studies in Slovenia (9) and Greece (1), the results of our studies indicated no case of malignant lesions in the studied population while cases of squamous cell carcinoma and adenocarcinoma have been reported in other similar studies with various prevalence rates (6, 7, 17). Associations between oral mucosal lesions, alcohol and tobacco smoking have been illustrated in several studies (2, 4) and it has been indicated that oral lesions would increase with age in association with tobacco consumption and denture use (24). In contrast, among our participants there was not a significant difference toward tobacco consumption between participants with and without oral lesions (P = 0.85) and oral lesions were more common in the age group of 30-40 years. Other factors such as trauma, medications and oral and dental hygiene have been found to play a role in oral mucosal changes and diseases (25). No statistically significant difference was confirmed between our participants with and without oral lesions regarding smoking (cigarette and tobacco), opium consumption, medication and oral and dental hygiene (use of toothbrush, toothpick, dental floss, mouthwashes, number of filled or decayed tooth) (P > 0.05). Our data has provided baseline information about epidemiologic aspects of oral mucosal lesions that can be valuable in organized national program targeting on oral health and hygiene in the society.
